# Comparison of Peptide Array Substrate Phosphorylation of c-Raf and Mitogen Activated Protein Kinase Kinase Kinase 8

**DOI:** 10.1371/journal.pone.0006440

**Published:** 2009-07-30

**Authors:** Kaushal Parikh, Sander H. Diks, Jurriaan H. B. Tuynman, Auke Verhaar, Mark Löwenberg, Daan W. Hommes, Jos Joore, Akhilesh Pandey, Maikel P. Peppelenbosch

**Affiliations:** 1 Department of Cell Biology, Section Immunology, University Medical Center Groningen, University of Groningen, Groningen, The Netherlands; 2 Laboratory for Experimental Internal Medicine, Academic Medical Center, Amsterdam, The Netherlands; 3 Department of Gastroenterology and Hepatology, Leiden University Medical Center, Leiden, The Netherlands; 4 Pepscan Presto, Lelystad, The Netherlands; 5 McKusick-Nathans Institute of Genetic Medicine, Johns Hopkins University, Baltimore, Maryland, United States of America; University of Texas MD Anderson Cancer Center, United States of America

## Abstract

Kinases are pivotal regulators of cellular physiology. The human genome contains more than 500 putative kinases, which exert their action via the phosphorylation of specific substrates. The determinants of this specificity are still only partly understood and as a consequence it is difficult to predict kinase substrate preferences from the primary structure, hampering the understanding of kinase function in physiology and prompting the development of technologies that allow easy assessment of kinase substrate consensus sequences. Hence, we decided to explore the usefulness of phosphorylation of peptide arrays comprising of 1176 different peptide substrates with recombinant kinases for determining kinase substrate preferences, based on the contribution of individual amino acids to total array phosphorylation. Employing this technology, we were able to determine the consensus peptide sequences for substrates of both c-Raf and Mitogen Activated Protein Kinase Kinase Kinase 8, two highly homologous kinases with distinct signalling roles in cellular physiology. The results show that although consensus sequences for these two kinases identified through our analysis share important chemical similarities, there is still some sequence specificity that could explain the different biological action of the two enzymes. Thus peptide arrays are a useful instrument for deducing substrate consensus sequences and highly homologous kinases can differ in their requirement for phosphorylation events.

## Introduction

Phosphorylation by protein kinases is involved in many facets of cellular regulation and plays an integral part of almost all signalling pathways by bringing about the transduction and amplification of various upstream signals [1]–[3]. Manning *et al* identified 518 putative protein kinase genes in humans, half of which were found to map to disease loci or cancer amplicons [4]. Most of these kinases are yet to be characterized and a substrate profile for each of these kinases would not only help decipher the complexity of these signalling cascades, but also enable the discovery of drug candidates to regulate their enzymatic activity.

Various methods have been described to predict phosphorylation sites by specific kinases: a database created by Kreegipuu *et al* from annotated phosphorylation sites found in literature[5] and Blom *et al* have used an artificial neural network method to predict eukaryotic phosphorylation sites [6]. Brinkworth *et al* have made use of the available crystal structures, molecular modelling and sequence analyses of kinases and substrates to predict the optimal substrate peptides [7]. Songyang *et al* have made use of an oriented peptide library to predict optimal substrates of protein kinases [8]. In this method, the kinase of interest was added to a soluble mixture of two and a half billion distinct peptides and then the phosphorylated peptides were separated from the bulk of non-phosphorylated peptides and sequenced to determine an optimal sequence for the kinase. Although a powerful and precise strategy, it is a very expensive and time consuming method.

Recent developments in array technology have now made it possible to make protein chips to study protein substrate interactions, and peptide chips for ligand-receptor interactions and enzymatic activities [9]–[15]. Very recently, Diks *et al* designed a novel peptide array to make descriptions of total cellular kinase activity [16]. In this approach, kinase substrates described in Phosphobase [17] were spotted on glass and incubated with cell lysates and radio active ATP. Subsequent phosphorylation of the peptides provided substrate phosphorylation profiles of LPS (lipopolysaccharide) -stimulated monocytes [16] and was also instrumental for the discovery of Lck (p56Lck) and Fyn (p59Fyn) as early targets of glucocorticoids [18]. It was also observed that many peptides were differentially phosphorylated. Many other studies using these arrays have been recently reported in the literature [19]–[22].

Importantly, in the study of Diks *et al* [16], purified PKA (Protein Kinase A) was employed for peptide array phosphorylation, and the results obtained suggested that peptide array phosphorylation was indeed capable of extracting the known optimal phosphorylation motif for PKA, although this possibility was not investigated in detail in this study. Encouraged by these results, we decided to explore the usefulness of peptide arrays for predicting optimal substrate sequences for kinases with as yet unknown substrate preferences. To this end, we used smaller arrays to study enzyme kinetics and determine experimental conditions of peptide array phosphorylation by purified kinases. These arrays, which were kindly provided by Pepscan Systems (The Netherlands), have 192 peptides spotted in duplicates. Diks *et al* have described the design of this array in great detail [16]. Subsequently, we employed a commercially available array, exhibiting 1176 Phosphobase database substrates to characterize the effects of two different MAP kinase kinase kinases (MAP3K). MAP3Ks form a part of a module which is classically activated by G-proteins. MAP3Ks on activation phosphorylate and activate a MAP kinase kinase (MAP2K; e.g., MEK) and finally activate a MAP kinase (MAPK; e.g., ERK). Thus, this MAP3K-MAP2K-MAPK module represents critical intermediate effectors that either positively or negatively propagate extracellular stimuli into cellular responses, such as differentiation, proliferation, and apoptosis. Two members from the MAP3K family, namely, c-Raf AND MAP3K8 (Mitogen activated kinase kinase kinase 8/c-Cot/Tpl-2) were used in this study.

c-Raf is a kinase important in human pathology, for instance, as a mediator of oncogenic Ras [23]–[26] or as an oncogene in its own right [27]–[29]. More recently c-Raf was also implicated as an essential mediator in chronic inflammation [30]. Analysis of the contribution of the individual amino acids in substrate peptides to total phosphorylation patterns enabled us to deduce a substrate consensus sequence for c-Raf. We were able to validate our results by using a different array containing 1024 peptide sequences derived from motifs in human proteins that are known to be phosphorylated. Analysis of the *in vitro* phosphorylation of this array yielded an almost identical preferential substrate sequence for c-Raf. Furthermore, we decided to exploit the possibility to use peptide arrays to predict kinase consensus sequences for deducing the preferential substrate peptide sequence of MAP3K8, a kinase which is homologous to c-Raf, but has a completely different function in cellular physiology, prompting the question whether both kinases share the same substrate preference or whether, despite the similarity in sequences both kinases have sufficient substrate specificity to account for the differences in biological function. MAP3K8 has been shown to participate in the transcriptional regulation of several important genes, including those for tumour necrosis factor alpha and IL-2 (Interleukin 2) [31]–[33]. MAP3K8 is also an integral component of signalling pathways that control the proteolytic processing of the NF-κB1 p105 protein [34] and is able to stimulate NF-κB-dependent transcription through the interaction and activation of the NF-κB-inducing kinase (NIK) [35]. Our study shows that peptide arrays are useful for deducing substrate consensus sequences and highly homologous kinases can differ in their requirement for phosphorylation events.

## Materials and Methods

### Reagents

Truncated constitutively active human MAP3K8 kinase and c-Raf were purchased from Upstate Biotechnology (Upstate Biotechnology, Lake Placid, NY). ^33^P-γ-ATP was purchased from Amersham Biosciences (Amersham Biosciences AB, Uppsala, Sweden). MEK^Ser218/222^/MEK2^Ser222/226^ antibodies were purchased from Upstate Biotechnology (Upstate Biotechnology, Lake Placid, NY). Lysis buffer was purchased from Cell Signaling Technology. Lysis buffer was supplemented with protease and phosphatase inhibitors, including 1 mM sodium fluoride, 1 µg/ml leupeptin, 1 µg/ml aprotinin, and 1 mM PMSF (Phenylmethanesulfonyl fluoride).

### Peptide Array design

The trial arrays consisting of 192 peptides were kindly provided by Pepscan Systems (Lelystad, The Netherlands). The full list of these substrates is listed elsewhere by Diks *et al* [16]. The array consisting of 1176 substrates was purchased from Pepscan systems (Lelystad, The Netherlands) and the design is described in detail on their website: http://www.pepscanpresto.com/index.php?id=27. We used a second array consisting of 1024 peptides based on known phosphorylated motifs in human proteins found in the Human Protein Reference Database (HPRD) [36] spotted in triplicate again made available by Pepscan Systems Briefly, a panel of known, phosphorylation motifs derived from different signalling cascades were selected from the proteins annotated in HPRD. Full list of peptides is available under license from the manufacturer's website. This is in contrast to the 1176 array, which used an unbiased set of amino-acid motifs that could be phosphorylated.

### Enzyme kinetics

Trial peptide arrays consisting of 192 peptide substrates were used to test enzyme kinetics. 5 µg/ml of purified active MAP3K8 was incubated with trial peptide arrays for 1, 5, 10, 20, 30, 45, 60 and 120 minutes.

### In vitro kinase assays

*In vitro* kinase assays were used according to the instructions of the manufacturer (Upstate Biotechnology, Lake Placid, NY). Active MAP3Ks were diluted in an Mg/ATP mixture and recombinant inactive MEK was added and in vitro kinase assays were performed at 30°C for 20 minutes. Samples were dissolved in sample buffer, incubated at 95°C for 5 minutes, and analyzed on Western blot using an anti-phospho-MEK^Ser218/222^/MEK2^Ser222/226^ Antibody.

### Kinase profiling

Peptide arrays with 1176 different kinase pseudo-substrates were incubated with active c-Raf and MAP3K8 incubation mix (end concentration of 5 µg/ml active MAP3K8 kinase and 2 µg/ml of active c-Raf, 8% glycerol, 0.5 mM ATP, 10 mM MgCl_2_, 0.05% v/v Brij-35, 25 µg/ml BSA (Bovine Serum Albumin)) and 30 µCi ^33^P-γ-ATP, at 37°C for 60 minutes in a humidified oven. The slide was then washed twice with PBS (+0.1% Triton X-100), 2M NaCl and demineralised H_2_O and dried with N_2_ gas. Only active c-Raf was incubated with the peptide array consisting of 1024 pseudo-substrates and the same washing steps were carried out.

### Peptide Array analysis

After drying, the glass slides were exposed to a phosphor imager plate for 72 hours. Acquisition of the peptide array was performed using a phosphor-imager (Storm™, Amersham Biosciences, Uppsala, Sweden). The level of incorporated radioactivity, which corresponds to the phosphorylation status, was quantified by Scanalyze. (http://rana.lbl.gov/EisenSoftware.htm) and exported to a spreadsheet program (Microsoft Excel 2002, Microsoft, Redmond, WA, USA). The relative contribution of each individual amino acid at each individual position was calculated and corrected for the relative abundance of that amino acid at that position relative to the central serine, threonine or tyrosine residues and the respective consensus sequences were generated.

### Difference between the two array designs

In order to determine how different the two arrays were from each other, we created a single sequence of all the peptide substrates on the 1024 array, separating each by ten gaps (the letter ‘X’ was used to create gaps). A set of hundred random peptides from the 1176 array design were generated using Microsoft Excel. (Microsoft Excel 2002, Microsoft, Redmond, WA) and each of these peptides were aligned individually against the single sequence obtained from the 1024 array design using MultAlin [37] with their default parameters. The number of identical amino acids were calculated for each of these hundred peptides and averaged to obtain an approximate estimation of similarity between these two array designs.

## Results

### Enzymatic characteristics of peptide array phosphorylation by purified enzymes

**Figure 1:**
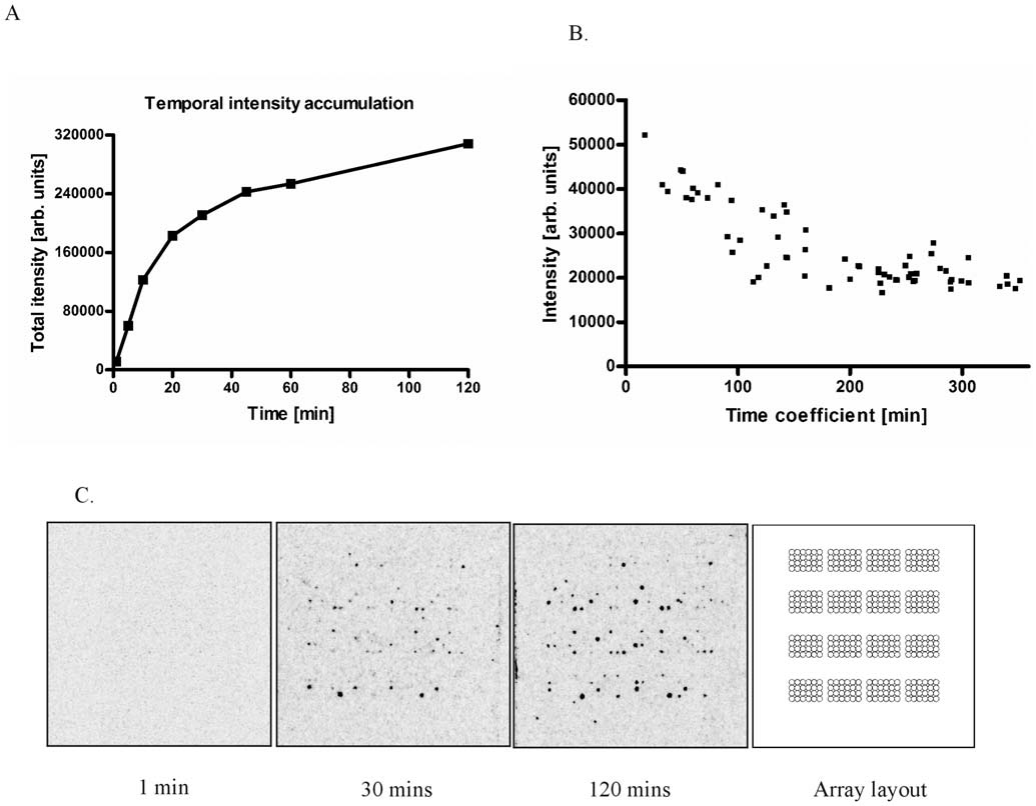
Determination of Enzyme Kinetics (MAP3K8). A. Plot showing the phosphorylation of various substrates at 1, 5, 10, 20, 30, 60 & 120 min. time points. B. Time coefficient plotted against intensity. C. Images of trial arrays at 1 minute, 30 minute and 120 minute time-points showing the steady increase in phosphorylation level. An overlay for array orientation is also shown.

We set out to evaluate the usefulness of peptide arrays for deriving consensus substrate sequences for kinases. To extract useful information from the phosphorylation of peptide arrays by kinases, it is important to ensure that such phosphorylation conforms to the Michaelis-Menten laws of enzyme kinetics. Hence, we decided to perform a series of initial experiments using active MAP3K8 on trial arrays consisting of 192 peptide substrates investigating array phosphorylation in the temporal domain. Figures 1A and IC show that a subset of substrate peptides displays increased phosphorylation at 1, 5, 10, 20, 30, 45, 60 & 120 minute time points, when incubated with 5 µg/ml of MAP3K8. A steady increase in phosphorylation intensities is seen till it reaches a steady state at the 60 minute time point, indicating that the peptide substrate levels only become a limiting factor after a 1 hour treatment. Figure 1B depicts a plot of the time coefficient deduced from the temporal results for peptide phosphorylation. The results suggest that a minimum of 30 minutes is required for efficient phosphorylation. Interestingly, the majority of substrates did not appear to be capable of undergoing phosphorylation by MAP3K8 at all, thus the MAP3K8 enzyme is not capable of catalyzing the phosphorylation of any given peptide and phosphorylation by this enzyme appears to exhibit qualitative characteristics: even prolonged incubation times do not yield detectable phosphorylation of unfavourable peptides.

### Generation of a putative c-Raf substrate consensus sequence

**Figure 2:**
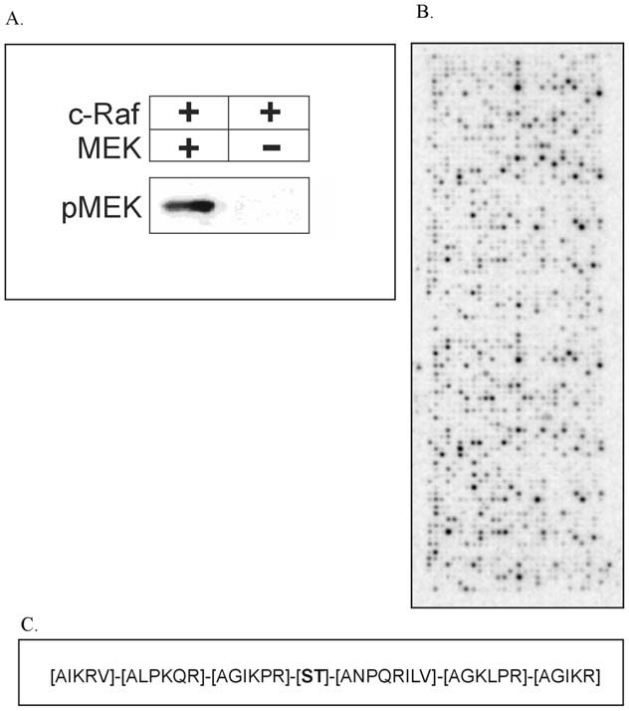
Analysis of phosphorylation of 1176 peptide array by c-Raf. A. *In vitro* phosphorylation of MEK by c-Raf. The capacity of purified c-Raf for in vitro phosphorylation studies was examined by incubating purified c-Raf with MEK and detected using MEK^Ser218/222^/MEK2^Ser222/226^ antibodies. B. c-Raf phosphorylation of 1176 peptide array. Phosphorylation of the 1176 peptide array, spotted in duplicate, on incubation with c-Raf and ^33^P-γ-ATP for one hour shows differential phosphorylation of the various substrate peptides demonstrating that peptide sequences confer specificity to c-Raf-dependent phosphorylation. Further analysis was carried out to determine whether the primary sequence of the peptides employed revealed information as to the amino acid preferences of this enzyme for substrate phosphorylation. C. Consensus sequence of c-Raf substrates using 1176 array design. Consensus sequence determined for c-Raf substrates on analysis of peptide array data shows a strong selection for both hydrophobic and basic residues at the −3 position. A strong preference for leucine is seen at the −2 position. Proline and arginine are strongly preferred at the −1 position. An examination of the amino acid preferences C-terminal to the fixed phosphorylated residue reveals a bias towards aspargine compared to other residues at the +1 position. Also, acyclic and hydrophobic amino acids are seen at the +1 position with no preference for any particular group of amino acid at the +2 position. The +3 position shows a strong preference for basic residues.

The capacity of c-Raf for *in vitro* phosphorylation studies was examined by incubating it with MEK (Mitogen Activated Protein Kinase Kinase), a well established substrate. As evident from figure 2A, our c-Raf preparation was highly active on MEK and we decided to test its ability to phosphorylate peptides immobilized in an array format containing 1176 phosphobase-derived peptides (see materials and methods). A one hour incubation with c-Raf resulted in extensive peptide phosphorylation, with different peptides incorporating wildly different amounts of ^33^P, demonstrating that peptide sequences confer specificity to c-Raf-dependent phosphorylation (figure 2B). Subsequent analysis was performed to see whether the primary sequence of the peptides employed revealed information as to the amino acid preferences of this enzyme for substrate phosphorylation.

It is possible that a peptide could be phosphorylated at more than one residue, which would mean that a peptide that, for instance, is phosphorylated at two serine's adjacent to each other could result in a higher intensity than a peptide phosphorylated on one serine and this would mask that peptide which could have been left out of the analysis. Hence, only those peptides which had a single phosphorylable residue were considered, i.e. only those peptides which had a single serine, threonine or tyrosine residue at the central position. Of the 1176 peptides, 353 peptides which had a single serine, threonine or tyrosine residue were selected. (Supplementary data S1). These peptides were then aligned manually relative to the centrally fixed serine, threonine or tyrosine residue and ranked on the mean intensity of the duplicates for each spot. For deriving the consensus sequence using arrays with 1176 substrates, we considered only positions −3, −2, −1, 0, 1, 2 & 3 because not all peptides were 9 amino acids in length and also while aligning them based on a fixed central phosphorylation site, we did not have an equal distribution of amino acids at the −4 and +4 positions.

Furthermore, we have only selected peptides with cut-off intensities within 50% of the peptide with the maximum intensity (peptide LRRASLRG with intensity of 37482.5 arbitrary units) and the relative contribution of each individual amino acid at each individual position was calculated and corrected for the relative abundance of that amino acid at that position relative to the central serine, threonine or tyrosine. The resulting putative c-Raf consensus sequence is shown in figure 2C, whereas table 1 lists the detailed results of the contribution of each amino acid at each position. For c-Raf, a strong selection for both hydrophobic and basic residues is seen at position −3, namely isoleucine and lysine. A strong preference for leucine is seen at position −2 with some preference for proline and arginine. There seems to be a strong selection for proline and arginine at the −1 position. An examination of the amino acid preferences C-terminal to the fixed phosphorylated residue reveals a bias towards aspargine compared to other residues at the +1 position. Also, acyclic and hydrophobic amino acids are seen at the +1 position. There is no preference for any particular group of amino acids at the +2 position. The +3 position also shows a strong preference for basic residues.

**Table 1:** Relative weights of amino acids for c-Raf using 1176 array design.

Amino Acid	Position								
	−4	−3	−2	−1	0	1	2	3	4
**A**	1.09	0.75	0.75	0.87	*	1.60	1.13	1.30	0.00
**D**	0.00	0.00	0.00	0.00	*	0.00	0.00	0.00	0.00
**E**	0.00	0.00	0.00	0.00	*	0.00	0.00	0.00	0.00
**F**	3.69	0.00	0.00	0.00	*	0.00	0.00	0.00	0.00
**G**	0.00	0.00	0.00	1.66	*	0.00	1.07	1.40	0.00
**H**	0.00	0.00	0.00	0.00	*	0.00	0.00	0.00	0.00
**I**	0.00	1.73	0.00	0.32	*	1.38	0.00	1.78	0.00
**K**	0.57	1.66	0.88	0.83	*	0.00	0.39	1.42	1.79
**L**	0.69	0.00	2.27	0.00	*	1.62	1.66	0.00	1.13
**M**	0.00	0.00	0.00	0.00	*	0.00	0.00	0.00	0.00
**N**	0.00	0.00	0.00	0.00	*	2.55	0.00	0.00	0.00
**P**	0.00	0.00	1.36	2.03	*	1.54	0.94	0.00	0.00
**Q**	0.00	0.00	0.73	0.00	*	0.91	0.00	0.00	0.00
**R**	2.11	1.10	1.38	1.85	*	0.96	1.75	2.05	2.60
**S**	*	*	*	*	0.91	*	*	*	*
**T**	*	*	*	*	1.58	*	*	*	*
**V**	2.35	1.39	0.00	0.00	*	1.17	0.00	0.00	1.03
**W**	*	0.00	0.00	0.00	*	0.00	0.00	*	*
**Y**	*	*	*	*	0.36	*	*	*	*

* replaces all divided/0 values.

### Verification of the consensus sequence on a different array

**Figure 3:**
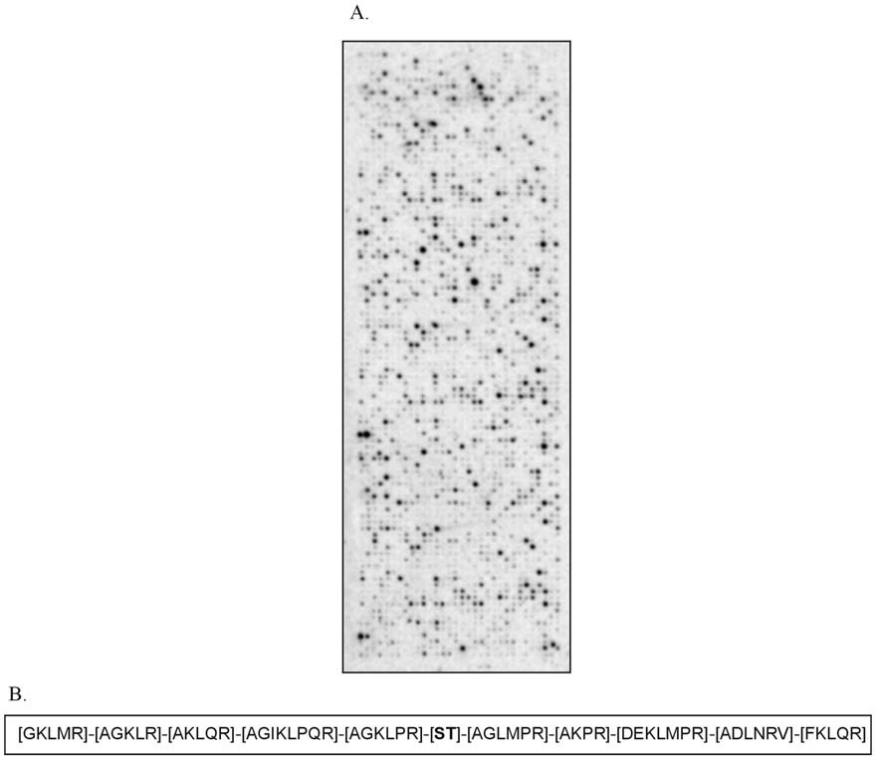
Analysis of phosphorylation of 1024 peptide array by c-Raf. A. c-Raf phosphorylation of 1024 peptide array. Phosphorylation of the custom made 1024 peptide array, spotted in triplicate, on incubation with c-Raf and ^33^P-γ-ATP for one hour shows differential phosphorylation of the various substrate peptides. B. Consensus sequence of c-Raf substrates using 1024 array design. Consensus sequence obtained from the 1024 peptide array shows a strong preference for arginine at −1,−4 and the −5 position while the −2 position shows a strong preference for a hydrophobic residue and no distinctive preference is seen at the −3 position. Analysis of the C-terminal position relative to the centrally fixed phosphorylated residue shows a very high preference for methionine besides an equal preference for other basic and hydrophobic amino acids at the +1 position. Arginine is preferred at the +2 and +4 positions while methionine and proline along with arginine are preferred at +3 positions. Hydrophobic residues are preferred over basic residues at +5.

If analysis of the contribution of each amino acid at each position in peptide array phosphorylation patterns yields meaningful results, it should follow that the analysis of phosphorylation of an array containing totally different substrate peptides, which on determination showed only five amino acids to be common, on average, between the two designs, should give a similar result. To test this hypothesis, we analyzed c-Raf-dependent phosphorylation of an array: consisting of 1024 peptides (figure 3A). Similarly, for the 1024 peptide array, peptides with single phosphorylation sites were selected for further analysis. (Supplementary data S2). The resulting putative c-Raf consensus sequence is shown in figure 3B, whereas table 2 lists the detailed results of the contribution of each amino acid at each position. There seems to be a strong preference for arginine at −1, −4 and the −5 position while the −2 position shows a strong preference for a hydrophobic residue and no distinctive preference is seen at the −3 position. Analysis of the C-terminal position relative to the centrally fixed phosphorylated residue shows a very high preference for methionine besides an equal preference for other basic and hydrophobic amino acids at the +1 position. Arginine is preferred at the +2 and +4 positions while methionine and proline along with arginine are preferred at the +3 position. Hydrophobic residues are preferred over basic residues at +5. Thus, totally different array designs yield similar c-Raf substrate consensus sequences, suggesting that this type of analysis is a valid tool for deducing kinase substrate preferences.

**Table 2:** Relative weights of amino acids for c-Raf using 1024 array design.

Amino Acid	Position										
	−5	−4	−3	−2	−1	0	1	2	3	4	5
**A**	0.00	1.17	1.58	1.79	2.28	*	1.30	2.36	0.00	1.09	0.00
**D**	0.00	0.00	0.00	0.00	0.00	*	0.00	0.00	1.72	1.24	0.00
**E**	0.00	0.00	0.00	0.00	0.00	*	0.00	0.00	0.79	0.00	0.00
**F**	0.00	0.00	0.00	0.00	0.00	*	0.00	0.00	0.00	0.00	3.17
**G**	1.56	1.78	0.00	1.45	2.02	*	1.38	0.00	0.00	0.00	0.00
**H**	0.00	0.00	0.00	0.00	0.00	*	0.00	0.00	0.00	0.00	0.00
**I**	0.00	0.00	0.00	2.54	0.00	*	0.00	0.00	0.00	0.00	0.00
**K**	2.30	1.86	1.92	1.45	0.84	*	0.00	1.17	1.07	0.00	0.96
**L**	0.70	0.64	1.27	1.60	0.68	*	1.68	0.00	0.74	1.16	2.20
**M**	1.60	0.00	*	0.00	0.00	*	3.12	0.00	2.36	0.00	0.00
**N**	0.00	0.00	0.00	0.00	0.00	*	0.00	0.00	0.00	1.51	0.00
**P**	0.00	0.00	0.00	0.97	1.33	*	1.09	1.45	2.12	0.00	0.00
**Q**	0.00	0.00	1.47	1.68	0.00	*	0.00	0.00	0.00	0.00	1.14
**R**	2.78	3.34	1.32	0.41	2.38	*	2.26	2.83	2.08	3.11	1.64
**S**	*	*	*	*	*	0.99	*	*	*	*	*
**T**	*	*	*	*	*	1.89	*	*	*	*	*
**V**	0.00	0.00	0.00	0.00	0.00	*	0.00	0.00	0.00	1.15	0.00
**W**	*	*	*	0.00	*	*	0.00	0.00	0.00	*	0.00
**Y**	*	*	*	*	*	0.00	*	*	*	*	*

* replaces all divided/0 values.

### c-Raf and MAP3K8 kinases are highly homologous but have substantially different substrate preferences

**Figure 4:**
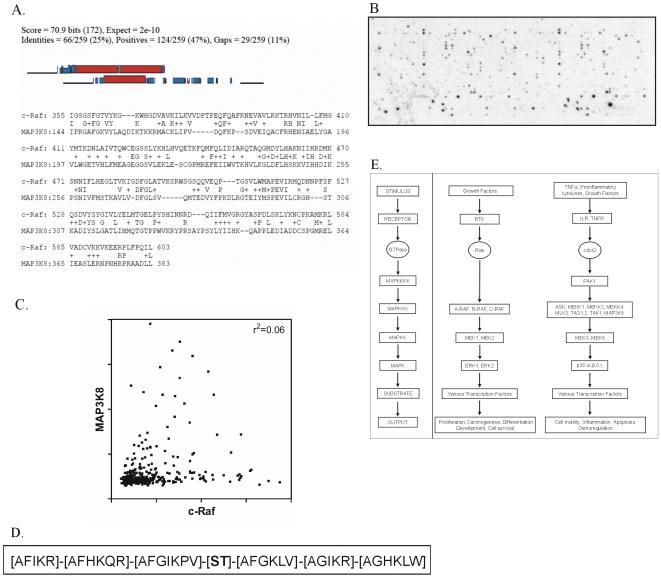
Analysis of phosphorylation of 1176 peptide array by MAP3K8 and comparison against c-raf. A. Alignment of c-raf against MAP3K8. Alignment of c-Raf with MAP3K8 using BLAST shows considerable homology between their kinase domains. B. Phosphorylation by MAP3K8 of 1176 peptide array. Specific incorporation of radioactivity into different peptides was observed after incubating the 1176 peptide array with MAP3K8 and ^33^P-γ-ATP. C. Comparison of c-Raf and MAP3K8 peptide phosphorylation. A correlation plot between substrate phosphorylation of c-Raf and MAP3K8 (using the 1176 peptide array design), which indicates that despite the highly similar primary sequences both enzymes have different substrate preferences. D. Consensus sequence of MAP3K8 substrates using 1176 array design. Consensus sequence for MAP3K8 shows preferences for phenylalanine at −3, −2, −1 and +1 positions and lysine at all six positions, which is similar to c-raf that has a preference for arginine instead of lysine at all positions as shown in figure 2C. E. Flow chart depicting the MAPK cascade and the roles of various kinases including c-Raf and MAP3K8 within these cascades.

Subsequently, we addressed the question whether the various MAP3Ks, which share substantial sequence homology (figure 4A) in their kinase domain, have identical or different substrate specificities. To this end, the 1176 array was incubated with active MAP3K8. As evident from figure 4B, again specific incorporation of radioactivity into different peptides was observed. Figure 4C shows a correlation plot between substrate phosphorylation of c-Raf and MAP3K8, which indicates that despite the highly similar primary sequences both enzymes have different substrate preferences.

### Analysis of MAP3K8 substrate preferences

Subsequent analysis, however, of the importance of the individual amino acids relative to the central residue shows that the substrate preference of both kinases also displays substantial similarities, with preferences for arginine, isoleucine, lysine and alanine at −3, −2, −1 and +1 positions which are also preferred by c-Raf. However, a major difference between the two is the strong preference for phenylalanine at positions −1, −2, −3, +1 in MAP3K8. Another important difference is the absence of proline at the −2 and +1 positions in MAP3K8. (Figure 4D and Table 3).

**Table 3:** Relative weights of amino acids for MAP3K8 using 1176 array design.

Amino Acid	Position								
	−4	−3	−2	−1	0	1	2	3	4
**A**	2.37	0.76	1.40	1.57	*	2.52	0.80	1.50	0.00
**D**	0.00	0.00	0.00	0.00	*	0.00	0.00	0.00	0.00
**E**	0.00	0.00	0.00	0.00	*	0.00	0.00	0.00	0.00
**F**	0.00	3.21	2.81	2.13	*	3.36	0.00	0.00	0.00
**G**	0.00	0.00	0.00	0.57	*	1.12	0.62	0.97	0.00
**H**	0.00	0.00	2.42	0.00	*	0.00	0.00	4.87	2.18
**I**	0.00	2.25	0.00	1.54	*	0.00	1.31	0.00	0.00
**K**	2.65	2.10	2.15	2.31	*	1.01	2.32	2.27	2.45
**L**	0.58	0.00	0.00	0.00	*	0.58	0.00	1.80	0.70
**M**	0.00	0.00	0.00	0.00	*	0.00	0.00	0.00	2.86
**N**	0.00	0.00	0.00	0.00	*	0.00	0.00	0.00	0.00
**P**	0.00	0.00	0.00	1.03	*	0.00	0.00	0.00	1.84
**Q**	0.00	0.00	1.88	0.00	*	0.00	0.00	0.00	0.00
**R**	1.40	0.40	0.38	0.00	*	0.00	1.67	0.00	1.10
**S**	*	*	*	*	1.10	*	*	*	*
**T**	*	*	*	*	1.14	*	*	*	*
**V**	0.00	0.00	0.00	1.22	*	1.47	0.00	0.00	0.00
**W**	*	0.00	0.00	0.00	*	0.00	0.00	5.72	*
**Y**	*	*	*	*	0.00	*	*	*	*

* replaces all divided/0 values.

## Discussion

The predominance of phosphorylation as a regulator of cellular metabolism makes it of utmost importance to know kinase substrates for proper understanding of cellular physiology. Unfortunately, our understanding of kinase action does not yet permit the determination of kinase substrates based on the primary sequence of proteins. Indeed, if kinases with highly similar amino acid composition have similar or distinct substrate preferences remain unknown. Hence, empirical methods for determining kinase substrate sequences remain essential. We describe here a novel methodology for predicting kinase substrates, which makes use of a library of peptides, known to serve as phosphorylation motifs to determine a kinase substrate consensus phosphorylation sequence and employ this methodology for comparing substrates for c-Raf and MAP3K8 enzymatic activity respectively. c-Raf and MAP3K8 are two serine/threonine kinases which are associated with cellular transformation, but which are suggested to have divergent functions in cellular physiology despite their high sequence homology. Employing peptide arrays we derived consensus sequences for substrate phosphorylation based on the relative importance of multiple amino acids (except serine, threonine and tyrosine) carried out at each position and this sequence could then be used to search databases and predict possible substrates. We considered only peptides which had a single phosphorylation site as one of the concerns we had was that some peptides could also be phosphorylated on more than one spot and would thus add to the intensity generated from that peptide. However, as suggested by Diks *et al* [16], it is still not known whether two phosphorylation sites on a peptide are phosphorylated simultaneously by the kinase.

Confidence in our results was bolstered by the observation that two peptide arrays displaying different peptides yielded similar results. On comparing the consensus sequence derived from the 1176 array design:

[AIKRV]-[ALPKQR]-[AGIKPR]-[**ST**]-[ANPQRILV]-[AGKLPR]-[AGIKR] with that derived from the 1024 array design:

[GKLMR]-[AGKLR]-[AKLQR]-[AGIKLPQR]-[AGKLPR]-[**ST**]-[AGLMPR]-[AKPR]-[DEKLMPR]-[ADLNRV]-[FKLQR] one can see that practically the same group of amino acids are seen at positions −3 to −1, except at positions +1 and +3 with methionine appearing instead of aspargine and glycine, and methionine in place of glycine respectively. Surprisingly, with the 1176 array, two peptide motifs with a central tyrosine residue are among the substrates favoured by c-Raf, showing that serine/threonine restriction of this kinase is not necessarily absolute. Maybe this observation has physiological relevance. Phosphorylation of Y_340_ in c-Raf itself is important for association to MEK, it's most important substrate, thus auto-phosphorylation of c-Raf at this residue may contribute to its physiological function, but obviously further studies are essential for answering this question. We feel, however, that weighing individual amino acids in their contribution to overall peptide array phosphorylation seems a valid tool for determining consensus sequences.

Comparison of c-Raf with MAP3K8 ([AFIKR]-[AFHKQR]-[AFGIKPV]-[**ST**]-[AFGKLV]-[AGIKR]-[AGHKLW]) shows the same set of amino acids dominating except for the strong presence of phenylalanine at all 3 positions N terminal and the +1 position C terminal to the centrally fixed serine/threonine residue. Another difference observed is the dominance of lysine over arginine at all positions where basic residues are seen. Although these two kinases show very similar consensus/scaffold sequences, they seem to phosphorylate completely different sets of substrates as seen in figure 4C, showing that the few amino acids in the consensus sequences which differ between the two kinases are indeed important in defining the substrate profile for that kinase. Thus, our results show that the similarity in the primary sequences of both kinases is reflected in a chemically similar substrate preference, but the gradual differences that remain, when combined are substantial enough to ensure substrate specificity for the kinases in cellular physiology. In close agreement, divergent downstream targets in living cells for these two kinases have been described in literature e.g. MEK for c-Raf [38]–[40] and NIK for MAP3K8 [41].

c-Raf seems to have a strong preference for basic residues at the −3 position and hydrophobic residues at the +1 position relative the phosphorylated serine/threonine residue. The Protein kinase C (PKC), AKT kinase (Protein Kinase B), mammalian AMP-activated protein kinase, SNF1 (sucrose non-fermenting kinase 1), calcium/calmodulin-dependant kinase, phosphorylase kinase have similar preferences for basic residues at the −3 position and hydrophobic residues at the +1 position [42]–[46]. As these kinases are phylogenetically considered to be close to c-Raf it seems that, a common evolutionary origin of kinases has consequences for substrate specificity.

With the consensus sequences determined, a broad choice of possible substrates remains for both c-Raf as well as MAP3K8 in the human genome. To test this, we performed kinase restricted pattern searches using Scanprosite [47], employing patterns consisting of only the most weighted amino acids from tables 1 and 3 for c-Raf, namely, [IKRV]-[LPR]-[GPR]-[ST]-[AILNPV]-[AGLR]-[AGIKR] and MAP3K8, namely, [FIK]-[AFHKQ]-[AFIKV]-[ST]-[AFGV]-[IKR]-[AHKLW] and found 33 and 6 hits respectively. Many of these proteins are not likely to represent true intracellular substrates for these kinases. However, we did find some interesting candidates such as Ephrin type-B receptor 4, which is known to be phosphorylated but the kinase remains unknown and could thus be an interesting substrate for c-Raf. We also found sites within the Mast/stem cell growth factor receptor and ribosomal protein S6 kinase alpha 1 which are known sites of phosphorylation for PKC and ERK and could well be putative c-Raf substrates. Confidence in our method was further bolstered when the most well established substrate for c-Raf, MEK1 phosphorylated at Threonine 292 was found back with the pattern search. For the six hits found with the MAP3K8 substrate pattern, we found two interesting candidates, both similar to each other, namely, Myristoylated alanine-rich C-kinase substrate (MARCKS) and the other named MARCKS-related protein. Both these substrates were phosphorylated on serines by various kinases including PKCβ and Serine Threonine Protein Kinase N. This is an interesting result because MARCKS is known to be phosphorylated by a MAPK family member on the matched site, although the exact upstream kinase remains unknown [48]. It would prove interesting to see if MAP3K8 could directly phosphorylate this protein *in vivo*. Moreover, these substrates also had phenylalanine at the +1 position C terminal to the centrally fixed serine residue (FKKS**F**KL for MARCKS and KKFS**F**KK for MARCKS-related protein), which was one of the important differences seen between the c-Raf and MAP3K8 consensus sequences as described above. It is also interesting to note that BLAST [49] searches with the consensus substrates often yield many matches in which the central serine/threonine is replaced by a phosphorylation incapable amino acid, possibly a reflection of evolutionary pressure to avoid non-regulatory phosphorylation events. In addition, many other factors (such as the presence of scaffolding proteins, adaptor proteins or intracellular localization) will *in vivo* influence the extent to which a motif is really subject to phosphorylation by c-Raf or MAP3K8. In this sense, the consensus sequence determination is more suitable for determining the possible upstream regulatory kinases when an amino acid is known to undergo phosphorylation, rather than providing insights in the downstream targets of a given kinase. Nevertheless, analysis of possible downstream targets may be useful for hypothesis generation. In conclusion, we have developed a new method to generate consensus sequences using peptide arrays based on the relative intensity of amino acids seen at all the positions N and C terminal to the centrally fixed serine/threonine/tyrosine residue. The general utility of this method would be identification of new substrates and it also has an edge over the oriented and combinatorial peptide microarrays as it has peptides with known phosphorylation sites for different kinases.

## Supporting Information

Data S1 Peptides with single phosphorylatable residues considered for 1176 array analysis
(0.34 MB DOC)

Data S2 Peptides on 1024 array with single phosphorylation sites that were used for analysis
(0.13 MB DOC)
