# Supplementary material for: Comparison of Peptide Array Substrate Phosphorylation of c-Raf and Mitogen Activated Protein Kinase Kinase Kinase 8
Source: PLoS One. 2009 Jul 30;4(7):e6440. doi: 10.1371/journal.pone.0006440 (PMC2713828; doi:10.1371/journal.pone.0006440)
Supplement: Data S1 — Peptides with single phosphorylatable residues considered for 1176 array analysis (0.34 MB DOC) [file pone.0006440.s001.doc]

| **PEPCHIP SPOT NO.** | **SEQUENCE** |
| --- | --- |
| 556 | LRRA**S**LRG |
| 752 | LG**S**PLRRR |
| 909 | RRRR**S**VA |
| 360 | RRRRP**T**PA |
| 948 | LG**S**ALRRR |
| 301 | RRRRA**S**VA |
| 222 | KRP**S**IRAKA |
| 161 | PR**T**PGGRR |
| 982 | KRP**S**LRAKA |
| 714 | VRKR**T**LRRL |
| 175 | KRKQG**S**VRGL |
| 266 | FKRP**T**LRRV |
| 318 | VPR**T**PGGRR |
| 844 | G**S**RRR |
| 315 | VIKR**S**PRKR |
| 510 | RKQI**T**VR |
| 821 | APR**Y**PGGRR |
| 1040 | KR**T**LR |
| 171 | KRAKAK**T**AKKR |
| 1051 | RRRRAA**S**VA |
| 170 | KRRG**S**VPIL |
| 60 | RRA**S**L |
| 256 | RRA**S**I |
| 786 | KRP**S**NRAKA |
| 1017 | APR**T**PGGRR |
| 590 | KRP**S**QRAKA |
| 198 | KRP**S**RRAKA |
| 2 | KRP**S**VRAKA |
| 98 | RRL**S**I |
| 534 | LRRA**S**LGAF |
| 357 | KRKQI**S**VR |
| 1105 | RRRA**S**VA |
| 452 | RRA**S**F |
| 1118 | RKR**T**LRRL |
| 907 | RAAA**S**RARQ |
| 371 | KRAQI**S**VRGL |
| 570 | KRKQI**S**VRGL |
| 494 | RRRP**T**PAML |
| 582 | KRFG**S**KAHM |
| 1078 | RRA**S**R |
| 768 | KKKF**S**FKKP |
| 480 | RPPGF**T**PFR |
| 22 | KIQA**S**FRGH |
| 105 | RRR**S** |
| 553 | KQI**S**VRGL |
| 730 | LRRA**S**LGAA |
| 851 | RRV**S**V |
| 168 | VKRGI**S**GL |
| 27 | NRAI**T**ARRQ |
| 490 | RRF**S**V |
| 1150 | KRP**S**FRAKA |
| 586 | KRP**S**ARAKA |
| 954 | KRP**S**GRAKA |
| 1156 | KIQA**S**FRGH |
| 294 | RRG**S**V |
| 1092 | AAR**T**PGGRR |
| 372 | KKA**S**FKAKK |
| 1073 | AP**T**PGGRR |
| 531 | LRRA**S**LG |
| 190 | KRKQI**S**VRG |
| 681 | AVRR**S**DRA |
| 66 | KQI**S**VR |
| 1010 | AKRI**S**GKMA |
| 264 | APR**S**PGGRR |
| 676 | RPPGF**S**PFR |
| 852 | APF**T**PGGRR |
| 132 | KKQI**S**VR |
| 794 | EEEA**Y**GWMD |
| 290 | VRRI**S**GL |
| 226 | APA**T**PGGRR |
| 567 | KAKQI**S**VRGL |
| 195 | LRGR**S**FMNN |
| 68 | APR**T**AGGRR |
| 137 | RKQI**S**VRGL |
| 757 | KAKV**T**GRWK |
| 1079 | RFFG**S**DRGA |
| 981 | LRRA**S**VA |
| 902 | RAR**S**RKE |
| 111 | LRRA**S**LAG |
| 656 | APL**T**PGGRR |
| 376 | KKLG**S**KKPQ |
| 211 | IHQR**S**RKRL |
| 194 | KRP**S**ERAKA |
| 530 | KKKKA**S**VA |
| 418 | KRP**S**HRAKA |
| 1171 | LLRP**S**RRVR |
| 390 | KRP**S**DRAKA |
| 499 | RKA**S**RKE |
| 300 | LRRN**S**I |
| 468 | DLFG**S**DDEE |
| 649 | AKDA**S**KRGR |
| 26 | KRP**S**KRAKA |
| 283 | RRAA**S**VA |
| 95 | RRKM**S**RGLP |
| 910 | VRFE**S**IRLP |
| 525 | VVGG**S**LRGA |
| 699 | RRA**S**VA |
| 871 | RKR**S**RKA |
| 303 | RKEI**S**VR |
| 338 | LRRA**S**VAQL |
| 858 | AAA**S**FKAKR |
| 535 | PRKG**S**PRKG |
| 87 | RRKA**S**GP |
| 184 | LEKK**Y**VRRD |
| 435 | GRRQ**S**LIED |
| 695 | FKK**S**FKL |
| 967 | KKRL**S**VERI |
| 827 | GRPI**T**PPRN |
| 895 | RRA**S**LG |
| 239 | GRRQ**S**LIQD |
| 1080 | PR**T**PGGRR |
| 265 | DGER**Y**DEDE |
| 892 | LRRG**S**LG |
| 1088 | LRRA**T**LG |
| 356 | PKDP**S**QRRR |
| 102 | RRRQ**S**VLNL |
| 548 | PEGD**Y**EEVL |
| 682 | VKRG**S**GL |
| 118 | RKR**S**AKE |
| 91 | LRRA**S** |
| 402 | EEEL**Y**LEPL |
| 500 | LRRW**S**LG |
| 328 | KKKA**S**VA |
| 353 | QEGL**Y**NELQ |
| 696 | LRRP**S**LG |
| 139 | LRRA**S**VA |
| 503 | RRA**T**PA |
| 32 | FFRR**S**KIAV |
| 711 | RAAH**S**IKGG |
| 296 | RKR**S**RAEA |
| 255 | NWHM**T**PPRK |
| 223 | NPGF**Y**VEAN |
| 160 | PKEV**Y**DVML |
| 1084 | KQI**T**VR |
| 686 | RRD**S**V |
| 100 | RKR**S**RAEF |
| 238 | EPAV**S**PLLP |
| 680 | RDPV**T**ENAV |
| 1060 | RRA**T**VA |
| 472 | RRP**T**VA |
| 479 | RKR**T**RKE |
| 751 | PLAG**S**PVIA |
| 466 | AALE**S**EDED |
| 914 | RENE**Y**MPMA |
| 291 | RRKG**T**DVNV |
| 725 | RKAA**S**VIAK |
| 297 | LKRA**S**LG |
| 707 | VGWP**T**VRER |
| 511 | VHNR**S**KINL |
| 496 | LGEG**T**P |
| 443 | DADE**Y**LIPQ |
| 64 | RRRA**S**QLKV |
| 923 | LRKA**S**LG |
| 263 | **S**GRGK |
| 766 | KRKQI**S**VGGL |
| 671 | DNPD**Y**QQDF |
| 272 | DLFG**S**DEED |
| 749 | KQG**S**GRGL |
| 1054 | AAA**S**FKAKK |
| 307 | VRKI**S**GL |
| 460 | APQ**T**PGGRR |
| 727 | LRRA**S**GG |
| 287 | LRRA**S**L |
| 829 | DLPG**T**EDFV |
| 1047 | RRP**S**V |
| 979 | LQDD**Y**EDMM |
| 1112 | VRR**S**DRA |
| 600 | IDKI**S**RIGF |
| 877 | AVRR**S**DAA |
| 862 | RVLE**S**FRAA |
| 1081 | KRRV**S**EV |
| 763 | KAAQI**S**VRGL |
| 926 | LRMF**S**FKAP |
| 334 | NRKP**S**KDKD |
| 386 | KRKN**S**ILNP |
| 947 | PKRG**S**GKDG |
| 864 | RRP**S**PA |
| 352 | PEND**Y**EDVE |
| 314 | RKR**S**AAE |
| 192 | QRRH**S**LEPP |
| 962 | KRKQI**S**VAGL |
| 441 | ERRK**S**HEAE |
| 1063 | DNID**S**QGRN |
| 207 | EQPG**S**DDED |
| 391 | LRGP**S**WDPF |
| 433 | EDVG**S**DEED |
| 976 | QNPV**Y**HNQP |
| 167 | PQPE**Y**VNQP |
| 242 | GDND**Y**IIPL |
| 93 | DRV**Y**VHPF |
| 1075 | RRKA**S**GPPV |
| 1048 | APE**T**PGGRR |
| 486 | VKRI**S**GL |
| 689 | LARA**S**LG |
| 461 | DFPL**S**PPKK |
| 1160 | KKDV**T**PVKA |
| 668 | RRP**T**PA |
| 1067 | RKR**S**RAE |
| 770 | PAPG**S**PEPP |
| 321 | RV**Y**VHPF |
| 46 | GDRF**T**DEEV |
| 424 | FEAR**Y**QQPF |
| 404 | IDME**S**QERI |
| 958 | KRRN**S**EFEI |
| 278 | AVDG**Y**VKPQ |
| 648 | RA**S**LG |
| 187 | KQPI**Y**IVME |
| 101 | LRAA**S**LG |
| 1087 | AKR**S**RKE |
| 237 | EDVG**S**DEEE |
| 335 | LRRA**S**PG |
| 1015 | EKAK**S**PVPK |
| 369 | KGQE**S**FKKQ |
| 438 | GDLQ**S**AEFH |
| 48 | EEPQ**Y**EEIP |
| 621 | GLGE**S**RKDK |
| 260 | RRRA**S**QLKI |
| 1056 | DKEV**S**DDEA |
| 1022 | ENPE**Y**LGLD |
| 1143 | PKKG**S**KKAV |
| 487 | RRKD**Y**PALH |
| 380 | LDPL**S**EPED |
| 1029 | ERHH**S**IDAQ |
| 641 | GGIR**S**LNVA |
| 514 | VNEL**S**KDIG |
| 465 | M**S**VEEV |
| 55 | EGVK**S**DQAE |
| 384 | LLPM**S**PEEF |
| 697 | RRVR**S**QEPG |
| 6 | LEKK**Y**VRRD |
| 1007 | NKQG**Y**KARQ |
| 51 | DAHK**S**KRQH |
| 180 | KKPP**T**PPPE |
| 1158 | KRKQI**S**GRGL |
| 94 | VRR**S**DAA |
| 234 | DIPE**S**QMEE |
| 591 | EDAE**S**EDEE |
| 29 | GKRQ**T**EREK |
| 289 | DRV**Y**IHPF |
| 826 | ENPQ**Y**FRQG |
| 31 | DEEE**S**EQGA |
| 493 | LHRA**S**LG |
| 683 | RRKD**T**PALH |
| 592 | ENAE**Y**LRVA |
| 800 | MQLK**S**EIKQ |
| 692 | LARN**S**I |
| 841 | RRAD**S**LQKN |
| 215 | IRQA**S**QAGP |
| 879 | RRKA**T**QVGE |
| 606 | ERRV**S**NAGG |
| 483 | LRAN**S**I |
| 839 | EGGR**T**VGAG |
| 672 | GDVK**Y**ADIE |
| 930 | LRRP**S**DQAV |
| 416 | EILN**S**PEKA |
| 779 | LMDK**Y**HVDN |
| 231 | EKRA**S**GQAF |
| 1119 | LRHA**S**LG |
| 1172 | QMAL**T**PVVV |
| 822 | DGNK**S**PAPK |
| 176 | KKDA**S**DDLD |
| 1176 | QRRR**S**LEPP |
| 655 | **S**DEEH |
| 1165 | PRRV**S**RRRR |
| 322 | VRRV**S**DDVR |
| 78 | RVRM**S**ADAM |
| 304 | PG**S**PQKR |
| 731 | PRKG**S**PKRG |
| 853 | DEKL**S**EILG |
| 819 | EKEI**S**DDEA |
| 975 | LMAP**S**EEDH |
| 23 | NKGA**S**QAGM |
| 625 | APVA**S**PAAP |
| 726 | LRRA**S**LDG |
| 1035 | EFPL**S**PPKK |
| 973 | QHLK**S**VMLQ |
| 408 | NAPV**S**ALGE |
| 1147 | PPEK**T**EEEE |
| 425 | GLLR**S**WNDP |
| 81 | AGDG**S**DEEV |
| 453 | AKGG**T**VKAA |
| 366 | KRRD**Y**LDLA |
| 45 | DMRQ**T**VAVG |
| 1071 | VGPD**S**D |
| 1122 | LRKV**S**KQEE |
| 634 | GDKK**S**KKAK |
| 227 | DEEE**S**EEAK |
| 720 | NRLQ**T**MKEE |
| 67 | **S**LKDH |
| 248 | DRRV**S**VAAE |
| 280 | GEIN**T**EDDD |
| 1144 | LEL**S**DDDD |
| 247 | DAGA**S**PVEK |
| 807 | NGDA**S**PAAA |
| 997 | EEPV**Y**EAEP |
| 5 | HRLL**T**LDPV |
| 889 | **T**AILE |
| 34 | EAAL**Y**KNLL |
| 797 | MGEA**S**GAQL |
| 991 | EQEE**Y**EDPD |
| 568 | KKAE**S**PVKE |
| 1149 | KAEE**Y**ILKK |
| 157 | QEPG**S**GPPE |
| 57 | RRAV**S**EQDA |
| 41 | EEDL**S**DENI |
| 153 | PAAV**S**EHGD |
| 854 | FGHN**T**IDAV |
| 921 | RHRD**T**GILD |
| 850 | QEKE**S**ERLA |
| 220 | EIRV**S**INEK |
| 49 | ERRL**S**LVPD |
| 549 | QEGD**T**DAGL |
| 796 | IAAD**S**EAEQ |
| 941 | PWRI**T**DNEL |
| 643 | EGNK**S**PAPK |
| 1042 | DAPD**T**PELL |
| 216 | NFDD**Y**MKEV |
| 1095 | VDEM**Y**REAP |
| 9 | MAEV**S**WKVL |
| 1141 | QEQE**Y**VQAV |
| 11 | EQQQ**T**EDEL |
| 666 | RVRI**S**ADAM |
| 833 | ERNL**S**FEIK |
| 427 | EKHH**S**IDAQ |
| 350 | PVPK**S**PVEE |
| 799 | IGRF**S**EPHA |
| 706 | RKQI**S**VR |
| 601 | MHRQE**T**VDA |
| 778 | KREA**S**LDNQ |
| 632 | ARVF**S**VLRE |
| 988 | HRQE**T**VDAL |
| 630 | ENQA**S**EEED |
| 454 | DDEI**T**QDEN |
| 700 | ADDE**Y**APKQ |
| 422 | ANDE**Y**FIRK |
| 561 | KAQE**Y**FNIK |
| 789 | HKIK**S**GAEA |
| 428 | GPAA**S**PAAA |
| 459 | **S**DEEV |
| 598 | EEEE**Y**MPME |
| 875 | KEAK**S**D |
| 693 | **T**IAVG |
| 675 | RKR**S**RKE |
| 580 | LKLA**S**PELE |
| 429 | AQAA**S**PAKG |
| 367 | PAPAVRA**S**DRA |
| 572 | KKKG**S**GEDD |
| 792 | HRQE**T**VEAL |
| 636 | EEKG**S**PLNA |
| 253 | RRAV**S**ELDA |
| 734 | LRRP**S**DQEV |
| 617 | GGVD**Y**KNIH |
| 661 | M**S**GDEM |
|  |  |

**Supplementary Data S1: Peptides with single phosphorylable residues considered for 1176 array analysis**

The peptides have been aligned by fixing the central serine, threonine or tyrosine resides.
