# Supplementary material for: Comparison of Peptide Array Substrate Phosphorylation of c-Raf and Mitogen Activated Protein Kinase Kinase Kinase 8
Source: PLoS One. 2009 Jul 30;4(7):e6440. doi: 10.1371/journal.pone.0006440 (PMC2713828; doi:10.1371/journal.pone.0006440)
Supplement: Data S2 — Peptides on 1024 array with single phosphorylation sites that were used for analysis (0.13 MB DOC) [file pone.0006440.s002.doc]

**Supplementary Data S2: Peptides on 1024 array with single phosphorylation sites that were used for analysis**

| **PEPCHIP SPOT NO.** | **SEQUENCE** |
| --- | --- |
| 365 | KKKIATRKPRF |
| 197 | KALPLSPRKRL |
| 443 | RKAKRSLAPRF |
| 845 | RRKAATMRERR |
| 732 | MRRQRSAPDLK |
| 953 | LRQLRSPRRAQ |
| 653 | RGKKKSGALVL |
| 581 | RRRRPTPAMLF |
| 1007 | GGRGGSRARNL |
| 110 | RLRQGTLRRDL |
| 907 | PGLRRSPIKKV |
| 624 | QERRKYLKHRL |
| 644 | IAVRKSRDKAK |
| 715 | RKKRISVKKKQ |
| 666 | QKIHISKKWGF |
| 396 | KKRKRSRWNQD |
| 151 | GPRRRSRKPEA |
| 342 | ALDFRTPRNAK |
| 194 | LDRFLSLEPVK |
| 1006 | KKPKKKSALLL |
| 434 | AKIQASFRGHM |
| 753 | PAARASKKILL |
| 453 | EAKRKSPKKKE |
| 746 | KKKKPSRLKGD |
| 161 | EILGDSQHADV |
| 734 | AEVLPSPRGQR |
| 144 | MRKKISNAQLQ |
| 887 | ALVRGTPVRGA |
| 974 | RLLGHSPVLRN |
| 834 | AMFPETLDEGM |
| 234 | RLRRDSKEANA |
| 831 | ENFDDYMKEVG |
| 213 | EEKQKSDAEED |
| 668 | LLEDDSDEEED |
| 475 | RDKEVSDDEAE |
| 491 | VHRDLSRDRPL |
| 793 | AENPEYLGLDV |
| 100 | EERKASGPPKG |
| 978 | EKRKNSILNPI |
| 997 | LLKLASPELER |
| 869 | LARRPTKGIHE |
| 505 | PLVQRGSANGL |
| 1023 | PVAPLSPARLQ |
| 102 | LIEPDTPGRVP |
| 691 | LLREASARDRQ |
| 836 | MHRRHTDPVQL |
| 289 | HPFLRRNSGA |
| 367 | FPAPQTPGRLQ |
| 468 | NLLPMSPEEFD |
| 805 | EEGEMYEDDEE |
| 546 | NVVPLYDLLLE |
| 502 | KIRRLSAAKQQ |
| 201 | HAVRESQVELR |
| 573 | QQQEVYGMMPR |
| 59 | GGDDIYEDIIK |
| 490 | QERRGSNVALM |
| 545 | PDEILYVNMDE |
| 676 | LMDNAYFAEAD |
| 888 | MRGILYAAPQL |
| 754 | LERNLSFEIKK |
| 275 | HVEDLYVEGLP |
| 210 | ELRRMSDEFVD |
| 446 | ILRKVSGHPNI |
| 683 | GLMQQQKSFR |
| 177 | DIEVESDEEQP |
| 331 | AERPLTQEELL |
| 17 | EGDEIYEDLMR |
| 809 | DKQVEYLDLDL |
| 553 | EDENLYEGLNL |
| 325 | LNRIQTQIRVV |
| 323 | LKGPGTPAFPH |
| 352 | NELKKKASLF |
| 69 | EPVQLTPDDED |
| 622 | MEEGQTQKGAF |
| 260 | PRQLNYIQVEL |
| 752 | RMVQLSPPALA |
| 225 | AGPALSPVPPV |
| 852 | IGDELYLEPLE |
| 221 | PEFPLSPPKKK |
| 640 | FFVIEYVNGGD |
| 961 | PPPQLSPFLQP |
| 748 | PALPQYPHING |
| 187 | DDLMLSPDDIE |
| 785 | QVKALYDFLPR |
| 808 | DDQEVYDDVAE |
| 321 | QELRKTFKEII |
| 326 | RGDVFTMPEDE |
| 508 | NIVLLSAEEKK |
| 73 | VDPMLTPEERH |
| 302 | LEDNDYGRAVD |
| 116 | FELAFSLDQPD |
| 462 | DAPPLSPFPHI |
| 171 | MIHNRSKINLQ |
| 789 | DADENYFINEE |
| 314 | RRAKHYVELLV |
| 12 | AEKPFYVNVEF |
| 83 | ENPNFTGKKME |
| 298 | EELAEYAEIRV |
| 112 | AEEKEYHAEGG |
| 917 | RARLLSDAANV |
| 235 | EVFDFSQRRKE |
| 9 | GLAEFYHPGQE |
| 495 | APKAPSKKEKK |
| 875 | LAHNVSKDNRQ |
| 906 | QLGPPSPVKMP |
| 276 | RNDQVYQPLRD |
| 45 | FDAHIYEGRVI |
| 600 | GVDGDYEDAEL |
| 1016 | KNKPRSPVVEL |
| 265 | VNLINYQDDAE |
| 1024 | ARVLGSEGEEE |
| 870 | PFRRHSWIAFD |
| 894 | PDVPRTPVGKF |
| 88 | ERGQEYLILEK |
| 916 | IIRQPSEEEII |
| 608 | EEEADSAFGDD |
| 1010 | MQRKLSVALAF |
| 149 | VGKIFSNVRIN |
| 27 | EPENDYEDVEE |
| 281 | KAVDGYVKPQI |
| 216 | QQLQLSPLKGL |
| 373 | PEKPKTPQQLW |
| 296 | KEDPIYDEPEG |
| 591 | LHRDKTPLHQK |
| 166 | AVHEDSGDEDG |
| 900 | PKEKDSPHMQD |
| 758 | ELEGISPDELK |
| 819 | RQGKDYVGAIP |
| 94 | GDAAETPPRPR |
| 982 | LEPQKSLGDEG |
| 474 | LIPQQSINEAI |
| 586 | KDDKLTPKIGF |
| 473 | LERQLSLEQEV |
| 678 | GGADDSAEEGD |
| 127 | HPPVLTPPDQE |
| 925 | LAQAFSDVILA |
| 1012 | KERWGSNELPA |
| 839 | EGAAATPERMA |
| 585 | VDAAVTPEERH |
| 661 | LKQGASPNVQD |
| 873 | EKPRLSFADRA |
| 511 | LEKRASGQAFE |
| 389 | IKQGEKHSA |
| 593 | VLRPETPRPVD |
| 122 | DPRLLSPQQPA |
| 257 | ADGMLTFAGPK |
| 850 | APAAPTPAAPA |
| 525 | RPDHIYDEPEG |
| 853 | IEQWFTEDPGP |
| 372 | DHIEVSDDEDE |
| 551 | RNPGFYVEANP |
| 479 | QAKVGSLDNVG |
| 381 | KEEPQTVPEMP |
| 633 | KLVQASEELLR |
| 891 | KEEKGSPLNAA |
| 794 | FIGEHYVHVNA |
| 58 | IKDDEYNPAQG |
| 512 | PIDMESQERIK |

The peptides have been aligned by fixing the central serine, threonine or tyrosine resides.
